# Supplementary material for: Proteomics Analysis of Andrographolide-Induced Apoptosis via the Regulation of Tumor Suppressor p53 Proteolysis in Cervical Cancer-Derived Human Papillomavirus 16-Positive Cell Lines
Source: Int J Mol Sci. 2021 Jun 24;22(13):6806. doi: 10.3390/ijms22136806 (PMC8268713; doi:10.3390/ijms22136806)
Supplement: Supplementary file 1 [file ijms-22-06806-s001.zip › ijms-1258209-supplementary/Supplementary Table S1.pdf]

Supplementary Table S1 Oligonucleotide used in this study.

Targets	Sequence 5′→ 3′	Ref.
Bax	F: CCCGAGAGGTCTTTTTCCGAG<br>R: CCAGCCCATGATGGTTCTGAT	1
HERC4	F: TGATAGATGGGGGATTGTCG<br>R: ACCCAGTGATTGGTGCTCAT	2
SMURF2	F: CGCTTGATCCAAAGTGGAAT<br>R: GGTTGATGGCATTGGAAAGA	3
GAPDH	F: TCATCAGCAATGCCTCCTGCA<br>R: TGGGTGGCAGTGATGGCA	4

References

1. Rada, M.; Nallanthighal, S.; Cha, J.; Ryan, K.; Sage, J.; Eldred, C.; Ullo, M.; Orsulic, S.; Cheon, D.-J., Inhibitor of apoptosis proteins (IAPs) mediate collagen type XI alpha 1-driven cisplatin resistance in ovarian cancer. *Oncogene* **2018,** 37, (35), 4809-4820.
2. Zhou, H.; Shi, R.; Wei, M.; Zheng, W.-L.; Zhou, J.-Y.; Ma, W.-L., The expression and clinical significance of HERC4 in breast cancer. *Cancer cell international* **2013,** 13, (1), 1-8.
3. David, D.; Jagadeeshan, S.; Hariharan, R.; Nair, A. S.; Pillai, R. M., Smurf2 E3 ubiquitin ligase modulates proliferation and invasiveness of breast cancer cells in a CNKSR2 dependent manner. *Cell division* **2014,** 9, (1), 1-18.
4. Wongjampa, W.; Ekalaksananan, T.; Chopjitt, P.; Chuerduangphui, J.; Kleebkaow, P.; Patarapadungkit, N.; Pientong, C., Suppression of miR-22, a tumor suppressor in cervical cancer, by human papillomavirus 16 E6 via a p53/miR-22/HDAC6 pathway. *PloS one* **2018,** 13, (10), e0206644.
